# Off-Target Effects of Recombinant Zoster Vaccine: Clinical Benefits, AS01B Adjuvant Immunobiology, and Observational Evidence

**DOI:** 10.3390/vaccines14100879

**Published:** 2026-10-03

**Authors:** Joseah Tang, Sudharshan Balaji, Hazel H. Oon

**Affiliations:** 1 Lee Kong Chian School of Medicine, Nanyang Technological University, Singapore 308232, Singapore; 2 National Skin Centre and Skin Research Institute of Singapore, Singapore 308205, Singapore; 3 Yong Loo Lin School of Medicine, National University of Singapore, Singapore 117597, Singapore

**Keywords:** autoimmune, cardiac, dementia, mortality, off-target, shingles, side effects, vaccine, virus, zoster

## Abstract

**Background/Objectives:** The recombinant zoster vaccine (RZV), Shingrix^®^, has shown great efficacy in protecting against herpes zoster. However, emerging evidence suggests that this RZV might provide benefits that extend beyond preventing varicella zoster virus (VZV) reactivation. Specifically, this scoping review explored the literature to investigate the reported non-zoster, off-target effects of the Shingrix^®^ RZV, and the possible mechanistic explanations for these effects. **Methods:** Keyword searches of PubMed database were conducted, along with manual searches of other relevant journals and reference lists of primary articles. **Results:** Evidence from the included studies demonstrated relationships between administration of RZV vaccination and reduced risk of dementia, Alzheimer’s disease and major adverse cardiological events (MACEs), while also conferring geroprotective effects. Mechanistic studies identified two possible causes, namely VZV suppression and AS01B adjuvant immunoregulation. Prevention of herpes zoster resulted in reduced risk of associated cognitive impairment and vasculopathy, reducing incidence of dementia and MACE. AS01B adjuvant was also linked to persistent monocyte reprogramming, and enhanced innate immune response and memory, known as “trained immunity”, which provided a plausible explanation for the observed off-target effects. Adverse events because of RZV administration were rare but included Guillain–Barré syndrome (GBS), autoimmune encephalitis and autoimmune flares occurring after vaccination. However, there is insufficient evidence to indicate a causal relationship between RZV administration and these rare immune-mediated adverse effects, and post-marketing surveillance still support a favourable safety profile. **Conclusions:** Current evidence suggests that RZV administration confers numerous off-target benefits via VZV suppression and the immunomodulatory effects of the AS01B adjuvant. As clinical evidence remains observational, further investigation of the pleiotropic effects of RZV should be carried out to explore its role in reducing all-cause mortality.

## 1. Introduction

Varicella zoster virus (VZV), also designated human herpesvirus 3 (HHV-3), is a neurotropic human alphaherpesvirus of the family *Orthoherpesviridae*, subfamily *Alphaherpesvirinae*. Primary infection with VZV causes varicella (chickenpox), after which the virus establishes lifelong latency in the sensory ganglia of the peripheral nervous system. Reactivation of latent VZV manifests as herpes zoster (shingles) [1]. VZV is transmitted predominantly via the airborne route and by direct contact with vesicular fluid, conferring a reproduction number (R_0_) of approximately 3.7–5.0 [2] and is highly contagious. To address the substantial burden of VZV disease, universal varicella vaccination programmes have been progressively initiated across multiple countries worldwide. Unlike earlier targeted strategies restricted to high-risk groups, these universal programmes mandate or recommend varicella vaccination for all eligible children, establishing broad herd immunity, effectively reducing community transmission, preventing outbreak frequency and decreasing hospitalisation rates [3].

The recombinant zoster vaccine (RZV), Shingrix^®^, attained broad global licensure after withdrawal of the live-attenuated Zostavax^®^, Merck, from the US since 18 November 2020 and withdrawal from the EU market on 1 June 2025 [4,5]. SKYZoster^®^, SK bioscience, is a live-attenuated single-dose zoster vaccine that was developed and licensed in Korea on 29 September 2017 and approved for use within Thailand (20 May 2020) and Malaysia (13 December 2022) [6]. Following the withdrawal of the Zostavax^®^ vaccines, Shingrix^®^ has become the preferred vaccine for herpes zoster prevention because of its superior efficacy compared with the live-attenuated vaccine [5,6].

The RZV, Shingrix^®^, is a sterile injectable suspension containing recombinant VZV glycoprotein E (gE) antigen adjuvanted with AS01B [7]. Beyond its intended role in herpes zoster prevention, increasing attention has been directed toward the possibility that RZV vaccination may exert broader non-specific or “off-target” effects on human health. A burgeoning body of observational and epidemiological studies have reported associations between RZV vaccination and reduced incidence of neurodegenerative conditions, including dementia and Alzheimer’s disease, as well as a decreased risk of adverse cardiovascular events and all-cause mortality, suggesting immunological benefits extending beyond the prevention of VZV reactivation. Similarly, growing interest in the biology of vaccine-induced immunomodulation has renewed attention surrounding heterologous vaccine effects, wherein a vaccine confers protection or benefits against pathogens and diseases unrelated to the original antigen target. Central to this is the concept of trained immunity [8], a form of innate immune memory mediated through epigenetic and metabolic reprogramming of myeloid cell progenitors, which enables a heightened and broadly reactive response to subsequent immunological challenges.

In the context of the RZV, the potential influence of its adjuvant systems on systemic inflammation and immunosenescence is of particular interest. The AS01B adjuvant system contained within RZV combines 3-*O*-desacyl-4′-monophosphoryl lipid A (MPL), a toll-like receptor 4 agonist, with the saponin QS-21 [7]. AS01B induces potent innate and adaptive immune activation, including dendritic cell stimulation, cytokine signalling and enhanced CD4+ T-cell responses. These immunologic properties have prompted speculation that some observed non-zoster benefits may extend beyond simple prevention of VZV reactivation and may instead reflect broader immunoregulatory or anti-inflammatory effects [9].

Given the growing clinical and scientific interest surrounding the potential non-specific effects of RZV, a critical synthesis of the available evidence is warranted. This semi-structured narrative review aims to evaluate current evidence regarding non-zoster outcomes associated with RZV, with particular focus on dementia and neurocognitive outcomes, cardiovascular disease, all-cause mortality and proposed mechanisms involving VZV suppression and AS01B-mediated immunoregulation.

## 2. Materials and Methods

A literature search was conducted using PubMed to identify articles related to off-target effects of VZV vaccination, using the following key terms and combinations: “recombinant zoster vaccine”, “Shingrix”, “herpes zoster vaccine”, “dementia”, “Alzheimer”, “cognitive decline”, “neurodegeneration”, “cardiovascular”, “stroke”, “myocardial infarction”, “MACE”, “mortality”, “non-specific effects”, “heterologous effects”, “trained immunity”, “immunoregulation”, “inflammaging”, “immunosenescence”, “geroprotection”, “AS01”, “AS01B”, “MPL”, “QS-21”, “adjuvant”, “dendritic cells”, “T cell response”, “adverse events”, “autoimmune”, “neurological”, “musculoskeletal”, “reactogenicity”.

Clinical studies, mechanistic studies, retrospective cohort studies and review articles published in English were analysed. Additional references were identified through manual screening of bibliographies from relevant papers and clinicaltrials.gov. The search was limited to articles published between 2021 and 16 June 2026, as the literature regarding the non-zoster and off-target effects of recombinant VZV vaccination emerged predominantly during this period.

## 3. Results

### 3.1. Dementia and Alzheimer’s Disease

Initial evidence linking herpes zoster vaccination with reduced dementia risk emerged from studies of the live-attenuated zoster vaccine (ZVL Zostavax^®^). Large retrospective cohort studies conducted in Wales and Australia have reported a consistent association between ZVL vaccination and subsequent reduced dementia risk. Across cohorts ranging from approximately 100,000 to over 9 million participants, these analyses consistently indicate an approximate 1–4% absolute reduction in probability of a new dementia diagnosis over a 7–8-year follow-up period in vaccinated individuals compared with unvaccinated controls. The consistency of findings across geographically distinct populations and multiple analytical frameworks strengthens confidence that the association is not confined to a single healthcare system or study design.

A natural experiment was conducted by Eyting et al., involving 282,541 people in Wales, using large-scale electronic health records (EHRs) data to compare individuals who were ineligible for ZVL because they were born immediately before the eligibility cut-off date of 2 September 1933, with those born immediately after who were eligible [10]. The authors reported a 3.5% reduced probability of a new dementia diagnosis over a follow-up period of 7 years, corresponding to a 19.9% relative reduction, with the observed protective effect being stronger among women than men [10]. Critically, the validity of this regression discontinuity design was supported by the absence of baseline differences across the cut-off regarding prior dementia, herpes zoster history, influenza or pneumococcal vaccination rates, or statin use [10,11].

Another natural experiment was conducted by Pomirchy et al., involving 101,219 people in Australia, similarly using eligibility for ZVL based on date of birth as the exposure criterion [12]. This study found that eligibility for ZVL (i.e., being born shortly after vs. shortly before 2 November 1936) was associated with a 1.8% reduced probability of receiving a new dementia diagnosis during the 7.4-year follow-up period [12]. Given that the interpretation of observational findings remains challenging because individuals who choose to receive vaccines often differ systematically from those who do not in terms of health-seeking behaviours, socioeconomic status and healthcare utilization, a phenomenon commonly referred to as healthy vaccinee bias [13], the natural experiments by Eyting et al. and Pomirchy et al. were specifically valuable because they substantially reduced this source of confounding by using eligibility for ZVL as the exposure criterion. Similarly, two other studies evaluating neurocognitive outcomes following ZVL administration consistently showed a reduced incidence of dementia across diverse geographical settings [14,15]. These natural experiments conducted in Canada and UK reported a reduced probability of receiving a new dementia diagnosis by 2.0% over a 5.5-year follow-up period [14] and an approximate 19% lower odds (Odds Ratio, OR, 0.808) of incident dementia, respectively [15], for those who are eligible for ZVL. Retrospective matched cohort studies in UK and South Korea also showed an association between ZVL administration and a lower risk of dementia diagnosis [16] and memory disorders [17], respectively.

More recent evidence suggests that the association between VZV vaccination and neurodegenerative outcomes may also vary according to host genetic susceptibility. Duan et al. found that ZVL receipt between ages 65 and 75 was associated with lower odds of subsequent Alzheimer’s disease in both the Health and Retirement Study (OR 0.55) and UK Biobank (OR 0.53), with stronger associations among carriers of the Alzheimer’s disease-associated NECTIN2 rs6859(A) allele (OR 0.43 and 0.51, respectively) [18]. A significant vaccination–genotype interaction was observed in the HRS cohort (interaction OR 0.48), although these findings predominantly reflect ZVL exposure and cannot presently be extrapolated to RZV [18].

While most early data were generated using ZVL, the introduction of the adjuvanted RZV provided an opportunity to examine whether this association extended to a more immunogenic vaccine platform. Taquet et al. addressed this question in a large, EHR- based natural experiment in the United States, including more than 200,000 people, comparing dementia incidence among those who received RZV immediately after versus before October 2017, the timepoint at which RZV uptake accelerated and the use of the live-attenuated vaccine waned [19]. In this setting, receipt of RZV was associated with a 17% increase in diagnosis-free time compared with recipients of live zoster vaccine, corresponding to approximately 164 additional dementia-free days over 6 years among those who eventually developed dementia [19]. Importantly, the association was consistent across most dementia subtypes, remained robust across multiple sensitivity analyses and was observed in both sexes [19], although the magnitude of benefit was 9% greater in women, echoing the findings of Eyting et al. for ZVL [10].

Similar results were obtained by a separate retrospective matched cohort study conducted by Rayens et al. of Kaiser Permanente Southern California members, which found that RZV vaccination was associated with a 51% lower risk of dementia with a HR of 0.49 [20]. The association remained significant when compared with an active Tdap-vaccinated comparator, with a HR of 0.73 [20]. Hayes et al. also supports a link between RZV and dementia prevention in a target trial emulation involving 509,926 participants, where the receipt of RZV was associated with a 24% reduction in the relative risk of dementia amongst older adults vaccinated with RZV (RR 0.76) over a 4-year follow-up period [21]. However, as negative control analyses suggest some residual confounding for this study, the findings support dementia prevention as a potential neuroprotective pleiotropic effect of RZV rather than establishing direct causality via the target trial emulation.

Further support was provided by Tang et al. in a retrospective cohort study of 5,402,678 immunocompetent adults using the Optum Labs Data Warehouse [22]. Dementia incidence decreased across vaccination categories, from 135.0 cases per 10,000 person-years among unvaccinated individuals to 108.2 and 99.1 cases per 10,000 person-years among partially and fully vaccinated individuals, respectively [22]. After adjustment for demographic characteristics, comorbidity burden, herpes zoster infection, antiviral use and prior ZVL receipt, one RZV dose was associated with an 11% lower hazard of dementia (HR 0.89), while completion of the two-dose series was associated with a 32% lower hazard (HR 0.68) [22]. This graded association according to vaccination status provides additional evidence supporting a potential dose–response relationship between RZV exposure and reduced dementia risk. Notably, the same analysis found that herpes zoster occurring before RZV vaccination was associated with increased dementia risk (HR 1.47), whereas antiviral treatment for HZ was associated with substantially lower risk (HR 0.42), providing epidemiological support for suppression of VZV reactivation as one potential contributor to the observed association between RZV and dementia [22]. Dos Reis et al. further supported an association between two-dose RZV and reduced dementia risk in 502,845 vaccinated and 1,005,690 unvaccinated Medicare beneficiaries [23].

Most recently, Rose et al. extended these findings in a preprint retrospective cohort study of approximately 1.5 million older adults from the U.S. National Clinical Cohort Collaborative. RZV vaccination was associated with a 29% lower hazard of incident late-onset Alzheimer’s disease with a HR of 0.71 over a maximum follow-up period of approximately 5 years [24]. Importantly, the protective association persisted irrespective of COVID-19 status, although SARS-CoV-2 infection significantly attenuated the magnitude of protection, suggesting that systemic viral infections may partially counteract the beneficial immunomodulatory effects of RZV [24]. The association remained robust after adjustment for healthcare-seeking behaviour and was not observed with pneumococcal vaccination, arguing against healthy vaccinee bias as the sole explanation [24].

Although several studies have reported associations between VZV vaccination and reduced dementia risk, the majority of available evidence derives from investigations of ZVL. Despite the predominance of ZVL-based evidence, the available RZV data are particularly intriguing because RZV induces substantially stronger and more durable cellular immune responses than ZVL and incorporates the potent AS01B adjuvant system. Consequently, whether the observed reduction in dementia risk is mediated primarily through prevention of VZV reactivation, broader immunological reprogramming, or a combination of both remains an important unanswered question that we will later discuss.

Currently, two main mechanisms are proposed to explain the association between VZV vaccination and reduced dementia risk. Firstly, prevention of VZV reactivation may reduce chronic neuroinflammation, cerebrovascular injury and other processes implicated in dementia pathogenesis. Additionally, the potent RZV contains immunostimulants that could contribute to the protective effect against dementia. These mechanisms are discussed in greater detail later in this review.

### 3.2. Cardiovascular Outcomes

RZV vaccination has also been linked to more favourable major adverse cardiovascular event (MACE) outcomes, including myocardial infarction (MI), stroke and coronary artery disease (CAD). Across multiple retrospective cohort studies involving 7000 to 4.9 million individuals from the United States, RZV vaccination was associated with reduced acute MI incidence, with HRs ranging from 0.72 to 0.84, as well as reduced stroke incidence, with HRs ranging from 0.58 to 0.87 over a follow-up period of 1–16 years.

A matched cohort study was conducted by Rayens et al., involving 102,766 adults aged ≥50 years who received two doses of RZV and 411,064 matched unvaccinated controls [25]. Over a mean follow-up period of 2.5 years, RZV vaccination was associated with significantly lower risks of hospitalized acute MI and hospitalized stroke, with HRs of 0.72 and 0.58, respectively [25]. Similarly, a retrospective cohort study conducted by Nguyen et al. within the United States using TriNetX, a large community-based database, enrolled 275,304 propensity-score matched pairs of adults ≤ 50 years with established atherosclerotic cardiovascular disease [26]. This study found that VZV vaccination was associated with a lower risk of MACE, MI, and all-cause mortality, with HRs of 0.54, 0.39 and 0.73, respectively [26]. In another propensity score-matched TriNetX cohort, RZV recipients also had a significantly lower risk of MI, with a rate ratio (RR) of 0.73, although no significant reduction in stroke was observed over 3 years [27]. Independent real-world analyses subsequently confirmed lower rates of MACE among RZV recipients [28], supporting the reproducibility of these findings across different healthcare databases.

Further evidence supporting cardiovascular benefits of RZV was provided by Kornelius et al., who conducted another retrospective cohort study in the United States involving 4.9 million patients with diabetes from 2006 to 2022 using TriNetX. This study investigated the association between VZV vaccination, including both RZV and ZVL, and risk of MACE in patients with diabetes [29]. Patients with diabetes mellitus were selected for investigation, as they are particularly susceptible to the compounded effects of VZV infection [29,30], having 2–3 times higher risk of cardiovascular events than in those without diabetes, underscoring the critical need for comprehensive strategies to mitigate these risks [29,31]. This study found that recipients of RZV demonstrated lower risks of MACE, CAD, stroke and all-cause mortality compared with unvaccinated controls, with HRs of 0.84, 0.78, 0.87 and 0.53 respectively [29]. Similar associations were observed among ZVL recipients, with reduced risks of MACE, CAD, stroke and all-cause mortality, evidenced by HRs of 0.81, 0.72, 0.90 and 0.58, respectively [29]. Interestingly, direct comparison between RZV and ZVL did not demonstrate statistically significant differences in overall MACE (HR 1.09), stroke (HR 0.96), or all-cause mortality (HR 0.99), although a modest increase in CAD risk (HR 1.16) was observed among RZV recipients [29]. These findings suggest that the observed cardiovascular benefits may be attributable to VZV vaccination more broadly rather than uniquely to the AS01B-adjuvanted recombinant platform, although direct head-to-head evidence remains limited. Kornelius et al. also demonstrated that the cardioprotective benefit of VZV vaccination is time-dependent, exhibiting the strongest risk reduction immediately post-vaccination before progressively attenuating [29]. This waning efficacy was evidenced by a sharp increase in HR for MACE during the first year, rising from 0.21 at 1 month to 0.35 at 2 months, 0.45 at 6 months and 0.57 at 12 months [29]. Over the extended follow-up period, the protective effect largely dissipated, with the HR for MACE reaching 0.93 at 5–10 years and 1.13 beyond 10 years [29].

Even when herpes zoster occurs, previous RZV vaccination and prompt antiviral therapy appears to attenuate the excess cardiovascular risk associated with herpes zoster infections. Xu et al. further examined cardiovascular outcomes among 112,637 adults who developed herpes zoster [30]. Prior RZV vaccination was associated with a 21% lower hazard of cardiovascular events (HR 0.79), while combined RZV vaccination and timely antiviral therapy conferred an even greater reduction (HR 0.61) [30]. Significant reductions were also observed for MI and stroke, supporting the concept that both prevention of herpes zoster and mitigation of breakthrough disease severity may reduce the excess cardiovascular risk associated with VZV reactivation [30].

However, in a 2025 TriNetX retrospective cohort study of patients with immune-mediated inflammatory diseases receiving JAK inhibitors, Shih et al. reported that RZV was not associated with a significant reduction in MACE over 5 years (HR 1.121), nor were there significant associations observed individually for MI (HR 0.948), ischemic stroke (HR 1.015) or heart failure (HR 0.956) [31]. The null association with MACE remained consistent across follow-up periods of 1–7 years [31]. Interestingly, RZV was nevertheless associated with a lower all-cause mortality (HR 0.610) by a similar degree as reported by Lin et al. (HR 0.606) [32], despite the absence of a detectable reduction in MACE [31]. This discordance argues against the cardiovascular event prevention as the sole explanation for the observed survival association and raises the possibility of broader effects of residual confounding, including healthy vaccinee bias.

Collectively, the evidence supports biological plausibility for cardiovascular benefits from VZV vaccination but remains insufficient to establish a causal cardioprotective effect, particularly for RZV. Herpes zoster itself is associated with a transient increase in vascular risk and observational ZVL studies suggest lower stroke incidence among vaccine recipients [25,29,30,31]. However, direct RZV evidence remains limited and is not uniformly supportive, with Shih et al. demonstrating no significant reduction in MACE despite an association with lower all-cause mortality [31]. Differences in vaccine formulation, study populations, cardiovascular risk profiles and residual confounding further limit cross-study comparisons. Thus, cardiovascular protection should presently be regarded as a potential non-target benefit of VZV vaccination rather than an established effect.

Proposed mechanisms behind the cardioprotective effects of VZV vaccination include the vaccine’s suppression of VZV infection, which can be linked to an increased risk of MACE, as well as its ability to modulate the immune response to reduce systemic inflammation, a key contributor to atherosclerosis and cardiovascular events. These mechanisms will be discussed in further detail later in this review.

### 3.3. Ageing and Immunomodulation

Emerging evidence also suggests that VZV vaccination may influence fundamental biological processes with ageing. Emerging epidemiological and mechanistic evidence suggests VZV vaccination may exert systemic geroprotective effects, potentially by modulating chronic inflammation, immune ageing and associated immune dysfunction.

Consistent with this, Kim et al. evaluated data from 3884 older adults in the United States Health and Retirement Study and found that ZVL recipients exhibited significantly lower inflammation scores (β = −0.14, *p* = 0.0027), slower epigenetic ageing (β = −0.19, *p* < 0.0001) and lower composite biological ageing scores (β = −0.18, *p* = 0.0002) compared to unvaccinated individuals, even after adjusting for demographic, socioeconomic and health-related factors [33]. ZVL vaccination was also found to be associated with lower epigenetic age acceleration as measured by DNA methylation-based ageing clocks, which are increasingly recognized as summary indicators of the cumulative ageing burden and are predictive of a range of later-life health outcomes, including mortality, frailty and chronic diseases [33]. This suggests that ZVL may influence overall core ageing processes, though the exact mechanisms remain unclear [33]. ZVL vaccination was also significantly associated with a lower score on a composite biological ageing measure that integrates signals across diverse biological systems, suggesting that ZVL’s effects are not confined to a single domain but may reflect broader biological recalibration [33]. These findings suggest that VZV vaccination may influence biological pathways implicated in healthy ageing, although it should be noted that vaccinations in this study largely involved ZVL rather than RZV.

Notably, the association between ZVL and biological ageing appear to be time dependent. Marked reductions in inflammation and improvements in adaptive immune function were observed predominantly among individuals vaccinated 4 or more years previously [33]. Similarly, associations with slower epigenetic and transcriptomic ageing remained detectable more than 4 years after vaccination [33]. These findings suggest that the biological effects of VZV vaccination may not be transient and raise the possibility of delayed or cumulative influences on immune ageing and molecular ageing pathways.

Mechanistic support for a potentially geroprotective role of RZV has been provided by recent immunological investigations. In a randomized clinical study conducted by Kilic et al. comparing younger and older adults, RZV elicited robust anti-gE antibody production, antigen-specific polyfunctional CD4+ T-cell responses and IFN-γ secretion across age groups, indicating an ability to overcome aspects of age-related immune decline [34]. Vaccination was associated with reductions in multiple circulating inflammatory mediators, particularly following the second dose [34]. Baseline inflammatory burden was inversely associated with subsequent vaccine-induced adaptive immune responses, supporting the concept that chronic low-grade inflammation contributes to immunosenescence and impaired immune function in older adults [34]. Hence, reduction of circulating inflammatory mediators by RZV could plausibly explain the reduced effect of age on immune decline.

Mechanistic studies into the AS01B adjuvant present in RZV were examined to further explore how RZV is able to preserve immune responsiveness despite age-related immune decline. RZV contains the AS01B adjuvant, which consists of two immunostimulants, the saponin QS-21 (*Quillaja saponaria* Molina, fraction 21) and the Toll-like receptor type 4 agonist, MPL (3-*O*-desacyl-4′-MPL) [3]. Exploration into the mechanisms of the immunostimulants by Welsby et al. demonstrated that at a cellular level, QS-21 undergoes cholesterol-dependent endocytosis by antigen-presenting cells and accumulates within lysosomes, where lysosomal destabilisation activates Syk- and cathepsin-B-dependent signalling pathways [35]. Additionally, AS01B-driven cytokine and innate immune cell responses are found to be transient, peaking predominantly within the first 24 h and largely resolving by day seven [36]. Importantly, MHC-II^high^ dendritic cells were shown to be necessary for efficient antigen presentation and subsequent T-cell priming, providing a mechanistic link between the transient innate response induced by AS01 and enhanced adaptive immunity [36]. Subsequent work further resolved this response at the cellular level, demonstrating recruitment of multiple conventional dendritic cell subsets to draining lymph nodes following AS01-adjuvanted vaccination, with cDC2 (conventional type 2 dendritic cells) and inflammatory cDC2 populations particularly effective at priming antigen-specific CD4+ T cells [37]. The functional consequence of this potent adjuvant activity is evident in human RZV recipients. In immunogenicity subcohorts of the ZOE-50 and ZOE-70 trials, 97.8% and 93.3% of vaccine recipients mounted humoral and gE-specific CD4+ T-cell responses, respectively, after the second dose [38]. Although responses subsequently declined, anti-gE antibody concentrations and gE-specific CD4+ T-cell frequencies remained 8.3- and 7.9-fold above baseline, respectively, at 36 months, demonstrating durable humoral and cellular immunity despite the predominantly transient initial innate response [38]. Collectively, these studies demonstrate how the AS01B adjuvant found in RZV results in potent innate immune activation by heightening downstream adaptive immune responsiveness.

Further mechanistic evidence was elicited from Johnson et al., who demonstrated that the increased responses of ex vivo gE-specific monocytes, DC cells and NK cells by RZV remained detectable for up to 5 years following vaccination and were unlinked to CD4+ T-cell responses [8]. Furthermore, sustained alterations in monocyte chromatin accessibility involving immune regulatory genes, such as TGFβ1, was also observed [35]. Additionally, sustained alterations in monocyte chromatin accessibility involving immune regulatory genes such as TGFβ1 was also observed [8]. These findings suggest that beyond heightened downstream adaptive immune responsiveness, RZV may also induce functional and epigenetic reprogramming of innate immune cells.

Together, these studies provide growing evidence to suggest that trained immunity is a biologically plausible framework through which RZV may contribute to protecting against age-related immune decline. However, clinical evidence remains limited, as much of the direct evidence relating biological ageing biomarkers to VZV vaccination derives from studies of ZVL rather than RZV.

Additionally, it is notable to consider the special population of immune-mediated inflammatory disease (IMID) patients undergoing immunotherapy with drugs like JAK inhibitors. Given that JAK inhibitors like TYK2 inhibitors raise VZV risk by disrupting interferon signalling, IMID patients may gain more proportionally from RZV’s protective downstream vascular and cognitive effects than the general population [39,40]. However, head-to-head and subgroup analyses on the IMID cohort remain an evidence gap.

### 3.4. Adverse Effects

RZV has demonstrated a favourable safety profile in large phase III clinical trials and post-marketing surveillance studies, with injection site reactions and transient systemic symptoms representing the most commonly reported adverse effects [41,42,43]. Rare immune-mediated adverse events have nevertheless been reported following RZV vaccination, such as isolated cases of Guillain–Barré syndrome (GBS), autoimmune encephalitis and autoimmune flares occurring after vaccination.

A case series cohort study involving 849,397 RZV-vaccinated individuals aged ≥65 years reported an increased risk of GBS among RZV vaccines compared with ZVL vaccines, with RR of 2.34 [44]. Similar findings were observed in self-controlled analyses, with RR of 2.84 [44]. Despite this relative increase in risk, the absolute attributable risk remained low, corresponding to approximately three additional GBS cases per million RZV doses administered [44]. Although the biological mechanism remains incompletely understood, proposed mechanisms include suppression of VZV reactivation, which can trigger GBS [44,45,46]. Further safety surveillance using electronic health record data conducted by Nelson et al. reported a similar safety profile with regards to GBS, as no sustained increased risk was detected [42]. However, due to the limited number of confirmed GBS cases observed, the study still reported uncertainty regarding potential associations between RZV and GBS [42,47]. Pharmacovigilance studies offer conflicting evidence. Multiple studies conducted using the Vaccine Adverse Event Reporting System (VAERS) and EudraVigilance databases have reported inconsistent GBS disproportionality signals for RZV [43,44,48,49,50], while a safety surveillance by Shu et al. noted no significant GBS signal in its disproportionality analysis [45]. However, Shu et al. conceded that uncertainty still persists regarding potential associations of RZV with GBS, warranting further investigation into GBS as a potential safety implication.

Besides GBS, other neurological signals included a case report that identified two individuals who developed autoimmune encephalitis less than 2 weeks after administration of RZV [51]. However, brain imaging and CSF serology were both unremarkable, and both patients recovered following pulse steroid therapy and plasmapheresis [51]. No clear mechanism has been identified either [51]. A separate case report by Wons et al. also described a case of serious and progressive neuropathy noted one week following reception of RZV and progressed until discharge 16 days post-admittance, with mild symptoms persisting [52]. However, exact diagnosis remains inconclusive, and no clear causal link has been established between RZV and the neurological signal. Another isolated case of myasthenia gravis (MG) was also identified in an 86-year-old Japanese woman approximately one week following RZV administration [53]. Being the only one of its nature, no definitive causal link could be established, and a possible hypothesis posited was RZV possibly unmasking or precipitating MG in a susceptible individual. However, no conclusive evidence was provided. In a real-world study on post-marketing safety for RZV in Shandong, China, Li et al. noted vestibular neuritis as a positive signal detected following data mining [54]. Despite this signal, however, Li et al. concluded that the safety profile of RZV remains within the expected limit.

A possible disproportionality signal for Henoch–Schönlein purpura (HSP) was identified by Wang et al. in their post-marketing surveillance study [55]. However, its exact cause remained unclear, with molecular mimicry by vaccine antigens and excessive activation of natural immune cells by the vaccine adjuvant being cited as potential explanations [55]. However, findings regarding HSP were inconclusive, and the study concluded that RZV nevertheless demonstrated a favourable safety profile but prompted continued monitoring for long-term adverse effects.

Autoimmune flares represent another potential adverse effect associated with RZV. In a pharmacovigilance analysis of the VAERS database involving 920 patients with pre-existing autoimmune diseases, 78 flare events were identified, corresponding to a reporting odds ratio (ROR) of 3.00 [56]. The strongest signals were observed among patients with systemic lupus erythematosus (SLE), multiple sclerosis (MS) and rheumatoid arthritis (RA), with RORs of 7.50, 5.61 and 5.03, respectively [56]. A total of 59% of reported flares occurred within three days of vaccination [56]. Proposed mechanisms include AS01-mediated activation of NLRP3 inflammasome and TLR4 signalling pathways, which have previously been implicated in autoimmune disease pathogenesis [57]. However, because the findings derive from spontaneous adverse-event reporting data, causal relationships cannot be established, and reporting bias remains possible.

Conversely, results from a randomised controlled trial substudy reported by Winthrop et al. demonstrated that satisfactory humoral and cell-mediated immune response to RZV were achieved in patients with rheumatoid arthritis receiving the Janus kinase inhibitor upadacitinib, without reports of any serious adverse events [58]. Over 60% achieved cell-mediated immune responses at all timepoints. Humoral responses were satisfactory at 87.8% of patients at week 16 and 71.4% at week 60 [58]. Furthermore, a retrospective matched cohort study found that RZV remained highly effective in preventing herpes zoster in patients with selected autoimmune disease and continued to offer substantial benefit to this patient population [59]. Furthermore, reassuring evidence has been provided by various studies that RZV is safe to use in populations with pre-existing RA [60,61], allogenic hematopoietic cell transplantation [62], gout [63], inflammatory bowel disease [64] rheumatic disease [65,66], systemic sclerosis [67], and various immune-mediated inflammatory diseases [68,69].

Additionally, in a prospective multicentre Korean study involving 1527 vaccine recipients, reactogenicity was greater following RZV than ZVL, particularly after the second RZV dose [70]. However, adverse-event frequency declined substantially by day 7 and no-life threatening vaccine-related events were observed [70]. Notably, no cases of GBS, autoimmune disease flares, clinically confirmed anaphylaxis, or fatal vaccine-related adverse events were identified during the 90-day follow-up period [70]. Similarly, a post-marketing surveillance of 10,392 herpes zoster vaccine doses in Australia reported a 6-fold increase in odds of a reported adverse event following immunisation in RZV (OR 6.44) compared to ZVL [71]. Nevertheless, only 0.6% of vaccinations lead to medical attention being sought, hence a favourable safety profile was still reported [71].

Ultimately, while rare immune-mediated adverse events have been reported, current evidence remains insufficient to establish definitive causal relationships for most proposed complications. Overall, the favourable benefit–risk profile of RZV demonstrated in clinical trials and real-world studies remains unchanged.

A comprehensive summary of the principal studies included in this review, including study design, population, comparator, effect estimates and main findings, is provided in Appendix A (Table A1).

## 4. Discussion

This study provides a comprehensive review of the protective and adverse off-target effects associated with RZV vaccination, including the clinical evidence surrounding each association. Here, the possible mechanisms behind these observed effects will be discussed in greater detail.

### 4.1. VZV Suppression Hypothesis

The VZV suppression hypothesis represents one of the leading and most widely hypothesised explanations for the observed off-target effects of RZV. It proposes that by preventing reactivation of latent VZV, RZV limits downstream pathological effects of VZV reactivation throughout the body, a process linked to the development of several conditions discussed in this review. VZV vaccination protects against herpes infections, which can cause dementia [72,73]. Reactivations of VZV have been linked to long-lasting cognitive impairment through vasculopathy [74,75], amyloid deposition and aggregation of Tau proteins [76], neuroinflammation [77,78,79,80] and cerebrovascular disease resembling that observed in Alzheimer disease, including small to large vessel disease, ischaemia, infarction and haemorrhage [77,78,79,80,81,82]. Additionally, there is a substantial body of evidence implicating the herpes simplex virus with the development of dementia [83,84], along with suggestive evidence that reactivation of VZV may lead to reactivation of the virus in the brain [85]. Although this causal link between herpes infection and dementia onset remains debated [86], such a pathway could account for the association between both RZV and ZVL vaccinations and reduced dementia risk, as well as the greater protective effect of RZV, given its superior protection against shingles [87].

Possible association between VZV infection and MACE onset has also been found. A primary mechanism believed to be at play is vasculopathy, where the virus directly infects and spreads from the nerve to the cerebral artery, eliciting transluminal inflammation with multinucleated giant cells and/or epithelioid macrophages, as well as pathological vascular remodelling [88,89]. These result in ischaemic infarction of the brain and spinal cord, aneurysm, subarachnoid and cerebral haemorrhage, carotid dissection and subsequently heightening the risk of stroke [88,89]. VZV infection may also contribute to elevated blood pressure due to the pain and stress associated with the condition [29]. Given that hypertension is a leading cause of stroke, this could further exacerbate the risk of stroke [90].

VZV vaccination also reduces chronic low-grade inflammation, “inflammaging”. Latent VZV remaining in the body can periodically reactivate, stimulating systemic inflammation by elevating inflammatory cytokines such as interleukin-6 (IL-6), tumour necrosis factor-α (TNF-α) and C-reactive protein (CRP) [33]. “Inflammaging” and ageing of the immune system, “immunosenescence”, are increasingly recognised as central features of ageing and major contributors to age-related diseases, including dementia, cardiovascular disease, frailty and mortality [34,91,92,93]. By simultaneously reducing systemic inflammation and promoting robust cellular immune responses, RZV may favourably modulate pathways implicated in biological ageing. These observations are consistent with previously illustrated epidemiological studies reporting reduced risks of dementia, cardiovascular events and all-cause mortality following VZV vaccination.

Interestingly, biological ageing studies did not identify significant associations between ZVL vaccination and established neurodegeneration biomarkers, including GFAP, neurofilament light chain, phosphorylated tau-181, or the Aβ42/40 ratio [33]. This observation suggests that any protective effect against dementia may occur indirectly through reductions in systemic inflammation, vascular injury, or immune dysregulation rather than through direct modification of classical neurodegenerative pathology [33].

### 4.2. AS01B Immunoregulation

Another possible mechanism for the off-target effects of RZV involves direct influence by the immunostimulants contained within the vaccine. In mice, QS-21 and MPL act synergistically to induce a high frequency of CD4+ T cells and additively to induce high antibody responses [7]. QS-21 activates inflammasomes in innate immune cells, particularly monocytes likely at the injection site and in peripheral macrophages within draining lymph nodes [7]. In human monocyte-derived dendritic cells, this induces a pro-inflammatory transcriptional programme, cytokine production and upregulation of antigen-presentation and costimulatory molecules, providing a mechanistic basis through which QS-21 can amplify downstream adaptive responses [71]. These monocytes then upregulate costimulatory molecules like CD86 and CD40 and produce inflammatory cytokines, including TNF-α, IL-1β and IL-6 [9]. This then stimulates NK cells and CD8+ T cells to release IFN-γ, which then stimulates activation and recruitment of both blood monocyte-derived and resident lymph node dendritic cells to take up and present gE to CD4+ T cells [67,68]. MPL works together with QS-21 to enhance the immune response to the co-administered antigen by producing IFN-γ, given that IFN-γ generally inhibits viral replication, also enhancing T cell responses and antibody isotype switching [36,94,95].

A study has also found that these aforementioned immune responses remain preserved in older adults despite immunosenescence [9]. Transcriptomic analyses demonstrated sustained activation of inflammatory pathways, including TNF and IL-1 signalling, even in elderly donors [9]. Age-related differences appeared more pronounced in metabolic pathways than in cytokine signalling, suggesting that AS01 can overcome aspects of age-related immune decline [9].

Network analysis identified upregulation of inflammatory regulators such as NFKBIA, JUN, and HIF-1ɑ [9]. HIF-1ɑ is notable because it is linked to trained immunity, a process involving epigenetic and metabolic reprogramming of innate immune cells that enhances future immune responsiveness [9]. AS01 also increased expression of autophagy-related genes involved in antigen presentation [9].

The temporal patterns observed in biological ageing studies may also provide support for this hypothesis. In older adults, favourable associations between VZV vaccination and inflammatory, epigenetic and transcriptomic ageing measures remained evident more than 4 years after vaccination [33]. Such persistence is difficult to explain solely through short-term immune activation and raises the possibility of longer-lasting immune reprogramming. One potential explanation is vaccine-induced epigenetic remodelling of innate immune cells through mechanisms similar to trained immunity, whereby prior immune stimulation produces long-lasting transcriptional and functional alterations [33]. Alternatively, sustained suppression of subclinical VZV reactivation may progressively reduce chronic inflammatory burden over time, as mentioned previously.

Together, these suggest that AS01B induces robust myeloid activation and may contribute to the unusually strong and durable immune responses observed with RZV, inducing potential broader immunomodulatory or off-target effects in ageing populations [9].

Consequently, multiple studies have proposed VZV-independent AS01B immunoregulation as a possible mechanism explaining RZV’s protective effects against dementia [10,12,19], cardiovascular events and ageing [29], though precise mechanistic pathways specific to each protective effect remain unclear.

In contrast, AS01B immunoregulation can also explain the aforementioned adverse off-target effects of RZV vaccination, specifically the autoimmune flares. Previous studies have confirmed that dysregulation of TLR4 function is closely associated with the pathogenesis of autoimmune diseases like SLE and RA [56,96,97]. In patients with SLE, an increase in transcobalamin 2 has been shown to amplify TLR4-mediated inflammatory responses, accelerating disease progression [56,98]. In patients with rheumatoid arthritis, HMGB1 interacts with TLR4 expressed on M1 macrophages, subsequently activating the STAT3 signalling pathway and promoting inflammatory responses [56,99]. Therefore, these findings point towards the possibility that AS01 induces or exacerbates autoimmune diseases [56,100].

The role of AS01 as a contributor to these observed effects can be further established by reported associations between Arexvy^®^, a respiratory syncytial virus (RSV) perfusion F3 (RSVPreF3) vaccine adjuvanted with the AS01E [101], and improved dementia and cardiovascular outcomes. In a propensity-score matched study comparing AS01-adjuvanted RZV and Arexvy^®^ with influenza vaccination, both the Arexvy^®^ and RZV cohort recipients demonstrated lower 18-month dementia risk [102]. No significant difference was observed between the two AS01-adjuvanted vaccines, and administration of both vaccines did not confer a clearly additive benefit [102]. Similarly, parallel cardiovascular associations have also been reported. In a large retrospective cohort of adults aged ≥60 years with pre-existing cardiovascular disease, Arexvy^®^ vaccination was associated with 65.0% effectiveness against RSV-related MACE and a more modest 20.4% effectiveness against overall MACEs [103]. As the two vaccines target unrelated viral antigens but share the AS01 adjuvant platform, these findings support the possibility that AS01-mediated immunoregulation contributes independently to the observed reduction in dementia and MACE risk. Comparable studies evaluating biological ageing outcomes after Arexvy^®^ have not been identified.

Mosquirix^®^ also incorporates the AS01 platform, specifically AS01E; however, because it is primarily administered in infancy and early childhood [104], comparable studies of dementia, cardiovascular outcomes and biological ageing are currently lacking and would not provide a directly comparable population to RZV recipients.

### 4.3. Clinical Implications

Although RZV is currently recommended primarily for prevention of VZV and its complications, accumulating evidence suggests that its clinical benefits may extend beyond protection against VZV reactivation. Observational studies have consistently reported associations between RZV or ZVL vaccination and reduced risks of dementia, cardiovascular events and all-cause mortality, while emerging mechanistic studies suggest possible effects on inflammaging, immunosenescence and broader immune regulation.

These findings may have important implications for a growing ageing global population increasingly burdened by chronic neurodegenerative and cardiovascular diseases. If causality can be established between the RZV vaccination and the observed protective effects, the public health impact of RZV will be significant. Such effects may also influence future cost-effectiveness analyses and vaccine policy decisions. Already, experts from the European Society of Cardiology (ESC) and American College of Cardiology (ACC) have acknowledged the protective benefits of RZV against cardiovascular events and suggest that vaccination can possibly be considered alongside other cardiovascular medications for clinical use in prevention of cardiovascular diseases [105,106].

However, current evidence remains insufficient to support the use of RZV specifically for prevention of dementia, cardiovascular disease or biological ageing. Most available data derive from observational studies and remain vulnerable to residual confounding despite increasingly sophisticated study designs. Consequently, RZV should continue to be recommended according to established indications, while potential off-target benefits should be regarded as promising but unproven.

Future research should prioritize prospective longitudinal studies, mechanistic investigations and comparative analyses capable of distinguishing the validity and relative contributions of VZV suppression and AS01B-mediated immunoregulation to RZV’s observed protective effects.

### 4.4. Limitations

Interpretation of the current literature is complicated by substantial methodological limitations. Much of the available evidence derives from retrospective observational cohorts and matched database analyses, which remain vulnerable to residual confounding, healthy vaccinee bias, healthcare utilization bias and differences in baseline healthcare status. Distinguishing true biological effects from epidemiologic artefacts therefore represents a major unresolved challenge within this field. Furthermore, the heterogeneity of study populations, outcome definitions, follow-up durations and analytical approaches complicates direct comparison across studies. The exclusion of non-English studies in the literature review may have also led to an omission of datasets and evidence from non-English healthcare systems.

## 5. Conclusions

Emerging evidence suggests that RZV may exert clinically relevant effects beyond prevention of VZV. Across multiple populations and study designs, VZV vaccination has been associated with reduced risks of dementia, cardiovascular events and all-cause mortality, as well as geroprotective effects, while mechanistic studies have identified potential influences on inflammation, immune ageing and broader immunological regulation. Although much of the epidemiological evidence remains observational, the consistency of findings across independent cohorts and natural experiments has generated increasing interest in the possibility that RZV may confer genuine off-target health benefits.

Two principal mechanistic frameworks currently dominate the field. The first proposes that suppression of VZV reactivation reduces downstream neurovascular and inflammatory consequences associated with dementia, cardiovascular and ageing-related diseases. The second suggests that AS01B-mediated activation of innate and adaptive immunity may induce broader immunoregulatory effects, potentially involving trained immunity, inflammaging reduction and partial reversal of immunosenescence. These mechanisms are not mutually exclusive and may act synergistically.

At present, the available evidence does not justify the use of RZV specifically for prevention of dementia, cardiovascular disease or ageing-related decline. Nevertheless, the accumulating epidemiological and mechanistic data suggest that the full benefits of RZV may extend substantially beyond herpes zoster prevention. Further prospective studies, mechanistic investigations and causal inference analyses will be required to determine whether these observations represent true biological effects and whether they can be leveraged to improve healthy ageing and long-term population health.

## Data Availability

All available data are included in the text.

## Abbreviations

The following abbreviations are used in this manuscript:

ACC	American College of Cardiology
CAD	coronary artery disease
cDC2	conventional type 2 dendritic cells
CRP	C-reactive protein
EHR	electronic health record
ESC	European Society of Cardiology
GBS	Guillain–Barré syndrome
gE	glycoprotein E
GFAP	glial fibrillary acidic protein
HIF-1ɑ	hypoxia-inducible factor 1-alpha
HHV-3	herpesvirus 3
HMGB1	high mobility group box 1
HR	hazard ratio
IBD	inflammatory bowel disease
IFN-γ	interferon-gamma
IL-6	interleukin-6
IMID	immune-mediated inflammatory disease
JAK	Janus kinase
JUN	Jun proto-oncogene, AP-1 transcription factor subunit
NFKBIA	nuclear factor-kappa-B-inhibitor alpha
NLRP3	nucleotide-binding domain, leucine-rich-containing family, pyrin domain-containing-3
MACE	major adverse cardiovascular events
MHC-II^high^	major histocompatibility complex class II-high expressing
MI	myocardial infarction
MPL	3-*O*-desacyl-4′-MPL
MS	multiple sclerosis
OR	odds ratio
QS-21	*Quillaja saponaria* Molina, fraction 21
RA	rheumatoid arthritis
ROR	reporting odds ratio
RR	rate ratio
RZV	recombinant zoster virus
SLE	systemic lupus erythematosus
STAT3	signal transducer and activator of transcription 3
TLR4	Toll-like receptor 4
TNF-α	tumour necrosis factor-alpha
TYK2	tyrosine kinase 2
VAERS	Vaccine Adverse Event Reporting System
VZV	varicella zoster virus
ZOE-50	zoster efficiency study in adults 50 years of age or older
ZOE-70	zoster efficiency study in adults 70 years of age or older
ZVL	live-attenuated zoster virus

## Appendix A

**Table A1:** Summary table of key studies and findings.

Outcome	Study	Vaccine	Design	Population	Comparator	Follow-Up	Effect Estimate	Main Finding
Dementia	[10]	ZVL	Natural experiment	Wales (282,541)	Eligibility cut-off	7 years	3.5% absolute reduction (95% CI 0.6–7.1)	19.9% relative reduction of probability of a new dementia diagnosis
Dementia	[12]	ZVL	Natural experiment	Australia (101,219)	Eligibility cut-off	7.4 years	1.8% absolute reduction (95% CI 0.4–3.3)	Reduced incident dementia
Dementia	[14]	ZVL	Natural experiment	Canada (232,124)	Eligibility cut-off	5.5 years	2.0% absolute reduction (95% CI 0.4–3.5)	Reduced incident dementia
Dementia	[15]	ZVL	Nested case–control	UK (228,223)	Unvaccinated	Variable	OR 0.808 (95% CI 0.657–0.993)	Approximately 19% lower odds of incident dementia
Dementia	[16]	ZVL	Matched cohort	UK (9,345,558)	Unvaccinated	Variable	HR 0.78 (95% CI 0.77–0.79)	Lower risk of dementia diagnosis
Dementia	[19]	RZV	Natural experiment	US (207,674)	VZL	6 years	17% longer diagnosis-free time	164 additional dementia-free days
Dementia	[20]	RZV	Matched cohort	US (329,000)	Unvaccinated	5 years	HR 0.49 (95% CI 0.46–0.51)	51% lower risk of dementia
Dementia	[24]	RZV	Retrospective cohort	US (1,479,580)	Unvaccinated	5 years	HR 0.71 (95% CI 0.69–0.74)	Lower risk of Alzheimer’s disease
Cardiovascular	[25]	RZV	Matched cohort	US (513,830)	Unvaccinated	2.5 years	MI: HR 0.72 (95% CI 0.59–0.88); stroke: HR 0.58 (95% CI 0.53–0.62)	Lower risks of MI and stroke
Cardiovascular	[27]	RZV	Matched cohort	US (7657)	Unvaccinated	3 years	MI: RR 0.73 (95% CI 0.55–0.96); stroke: RR 0.97 (95% CI 0.75–1.26)	Lower risk of MI but not stroke
Cardiovascular	[28]	RZV	Retrospective cohort	US (2,165,584)	Unvaccinated	30 days post- herpes zoster infection	MI: OR 0.82 (95% CI 0.74–0.92)	Lower risk of MI postinfection
Cardiovascular	[29]	RZV, ZVL	Retrospective cohort	US (4,903,424 with diabetes)	Unvaccinated; RZV vs. ZVL	Up to 16 years	RZV: MACE HR 0.84 (95% CI 0.76–0.91), CAD HR 0.78 (95% CI 0.69–0.88), stroke HR 0.87 (95% CI 0.77–0.99), all-cause mortality HR 0.53 (95% CI 0.48–0.58); ZVL: similar reductions, MACE HR 0.21 (95% CI 0.16–0.27) at month 1to 1.13 (95% CI 0.92–1.39) at year >10	Both RZV and VZL were associated with reduced cardiovascular events, with cardioprotective effects attenuating over time
Cardiovascular	[30]	RZV	Retrospective cohort	US (112,637 adults ≥50 years with herpes zoster)	No RZV; antiviral combinations	1 year	RZV alone: HR 0.79 (95% CI 0.67–0.94); RZV + antiviral: HR 0.61 (95% CI 0.53–0.72)	Prior RZV and antiviral therapy attenuated cardiovascular risk
Ageing	[33]	ZVL	Cohort	US (3884)	Unvaccinated	Variable	Reduced inflammation (β = -0.14, *p* = 0.0027); reduced epigenetic ageing (β = -0.19, *p* < 0.0001); reduced composite biological ageing (β = -0.18, *p* = 0.0002)	Reduced inflammation; slower molecular and biological ageing
Guillain–Barré syndrome	[44]	RZV	Case series cohort	US (849,397)	RZV vs. ZVL; self-controlled risk vs. control period	42 days	RR 2.34 (95% CI 1.01–5.41); self-controlled RR 2.84 (95% CI 1.53–5.27); attributable risk 3/million doses	RZV was associated with a small but increased short-term risk of GBS compared with ZVL; self-controlled analyses yielded similar results
Autoimmune flares	[56]	RZV	Pharmacovigilance analysis	US (920 with pre-existing autoimmune diseases)	Unvaccinated	Variable	Positive flare signal: ROR 3.00 (95% CI 2.40–3.75); SLE ROR 7.50 (95% CI 3.33–16.87); MS ROR 5.61 (95% CI 3.17–9.95); RA ROR 5.03 (95% CI 3.66–6.91)	RZV was associated with autoimmune flares in patients with SLE, MS and RA
Immunogenicity	[58]	RZV	Randomised controlled trial substudy	Global (93 with RA receiving upadacitinib)	RZV + upadacitinib vs. placebo + upadacitinib	60 weeks	Humoral response: 87.8% (95% CI 81.0–94.5%) at week 16, 71.4% (95% CI 61.8–81.1%) at week 60; cell-mediated response: >60% at all timepoints	RZV induced robust humoral and cell-mediated immunity without serious vaccine-related adverse events
Vaccine effectiveness	[59]	RZV	Retrospective matched cohort	US (145,874 with pre-existing autoimmune diseases)	Unvaccinated	Median 1.3 years (vaccinated) vs. 1.1 years (unvaccinated)	Overall vaccine effectiveness 66.3% (95% CI 61.4–70.7); herpes zoster incidence 4.3 vs. 12.9 per 1000 person-years	Two-dose RZV was effective in reducing herpes zoster incidence across six autoimmune diseases (RA, SLE, MS, IBD, psoriasis and psoriatic arthritis), with vaccine effectiveness ranging from 48.1% to 77.2%
Reactogenicity and safety	[70]	RZV, ZVL	Prospective multicentre survey	South Korea (1527)	ZVL vs. first-dose RZV; first-dose vs. second-dose RZV	90 days	Second-dose RZV had significantly higher adjusted odds of 9 solicited adverse events than first-dose RZV on Day 1; ZVL had significantly lower odds of 11 solicited adverse events than first-dose RZV. Differences largely resolved by Day 7. No fatal vaccine-related adverse events.	RZV was more reactogenic than ZVL, particularly after the second dose, but reactions were transient and no serious vaccine-related safety signals (including GBS, autoimmune flares, anaphylaxis or death) were observed during the 90-day follow-up.

## References

[1] Arvin A.M. (1996). Varicella-Zoster Virus. Clin. Microbiol. Rev..

[2] Marangi L., Mirinaviciute G., Flem E., Scalia Tomba G., Guzzetta G., Freiesleben de Blasio B., Manfredi P. (2017). The Natural History of Varicella Zoster Virus Infection in Norway: Further Insights on Exogenous Boosting and Progressive Immunity to Herpes Zoster. PLoS ONE.

[3] Adams L., Karachaliou Prasinou A., Trotter C. (2025). Modelling the Impact and Cost Effectiveness of Universal Varicella Vaccination in England. Vaccine.

[4] Centers for Disease Control and Prevention (2024). Shingles Vaccine Recommendations. https://www.cdc.gov/shingles/hcp/vaccine-considerations/index.html.

[5] European Medicines Agency (2025). Zostavax. https://www.ema.europa.eu/en/medicines/human/EPAR/zostavax.

[6] Baik S.H., Lee H., Kim H., Kim H.Y. (2025). Post-Marketing Surveillance of a Live Attenuated Herpes Zoster Vaccine (SKYZoster^®^) in Adults Aged ≥50 Years in Korea. Infect. Chemother..

[7] Heineman T.C., Cunningham A., Levin M. (2019). Understanding the Immunology of Shingrix, a Recombinant Glycoprotein E Adjuvanted Herpes Zoster Vaccine. Curr. Opin. Immunol..

[8] Johnson M.J., Crotteau M., Ghosh D., Vu T., Trinity L., Marches R., Ucar D., Levin M.J., Weinberg A. (2025). The Recombinant Zoster Vaccine Induces Trained Immunity in Monocytes through Persistent Downregulation of TGFβ. PLoS Pathog..

[9] Smith C.L., Richardson B., Rubsamen M., Cameron M.J., Cameron C.M., Canaday D.H. (2024). Adjuvant AS01 Activates Human Monocytes for Costimulation and Systemic Inflammation. Vaccine.

[10] Eyting M., Xie M., Michalik F., Heß S., Chung S., Geldsetzer P. (2025). A Natural Experiment on the Effect of Herpes Zoster Vaccination on Dementia. Nature.

[11] Eyting M., Xie M., Heß S., Geldsetzer P. (2023). Causal Evidence That Herpes Zoster Vaccination Prevents a Proportion of Dementia Cases. medRxiv.

[12] Pomirchy M., Bommer C., Pradella F., Michalik F., Peters R., Geldsetzer P. (2025). Herpes Zoster Vaccination and Dementia Occurrence. JAMA.

[13] Hosseini-Moghaddam S.M., He S., Calzavara A., Campitelli M.A., Kwong J.C. (2022). Association of Influenza Vaccination with SARS-CoV-2 Infection and Associated Hospitalization and Mortality Among Patients Aged 66 Years or Older. JAMA Netw. Open.

[14] Pomirchy M., Chung S., Bommer C., Strobel S., Geldsetzer P. (2026). Herpes Zoster Vaccination and Incident Dementia in Canada: An Analysis of Natural Experiments. Lancet Neurol..

[15] Lophatananon A., Mekli K., Cant R., Burns A., Dobson C., Itzhaki R., Muir K. (2021). Shingles, Zostavax Vaccination and Risk of Developing Dementia: A Nested Case-Control Study-Results from the UK Biobank Cohort. BMJ Open.

[16] Lophatananon A., Carr M., Mcmillan B., Dobson C., Itzhaki R., Parisi R., Ashcroft D.M., Muir K.R. (2023). The Association of Herpes Zoster and Influenza Vaccinations with the Risk of Developing Dementia: A Population-Based Cohort Study within the UK Clinical Practice Research Datalink. BMC Public Health.

[17] Oh J., Lee K., Yeo D., Cho J., Kim T.H., Lee J., Lee H., Woo H.G., Yon D.K. (2026). Herpes Zoster Vaccination and Cognitive Disorders in Older Adults. Alzheimers Dement..

[18] Duan H., Ukraintseva S., Holmes R., Wu D., Yashkin A.P., Akushevich I., Yashin A., Arbeev K. (2026). Towards Personalized Vaccine Repurposing for Alzheimer’s Prevention: Genotype-Specific Protective Association of the Shingles Vaccine with Odds of Alzheimer’s Disease in Participants of Two Large Cohort Studies. BMC Neurol..

[19] Taquet M., Dercon Q., Todd J.A., Harrison P.J. (2024). The Recombinant Shingles Vaccine Is Associated with Lower Risk of Dementia. Nat. Med..

[20] Rayens E., Sy L.S., Qian L., Ackerson B.K., Tubert J., Luo Y., Modha P.P., Calderon R.O., Chmielewski-Yee E., Oraichi D. (2026). Recombinant Zoster Vaccine Is Associated with a Reduced Risk of Dementia. Nat. Commun..

[21] Hayes K.N., Harris D.A., McConeghy K.W., Grove L.R., Joshi R., Han L., Davidson H.E., Chachlani P., Bayer T.A., Singh M. (2026). Dementia Risk After Recombinant Herpes Zoster Vaccination in Older Adults with a Recent Skilled-Nursing Facility Stay: A Target Trial Emulation. Ann. Intern. Med..

[22] Tang E., Ray I., Arnold B.F., Acharya N.R. (2025). Recombinant Zoster Vaccine and the Risk of Dementia. Vaccine.

[23] Dos Reis S., Tran P., Mohanty K., Amill-Rosario A., Johnson A., Ryan K., Alsdurf H., Oraichi D., Yun H. (2026). Reduced Risk of Dementia with Recombinant Zoster Vaccine in US Adults Age 65 or Older. Alzheimers Dement..

[24] Rose C.M., Liu S., Bush W.S., Haines J.L., Williams S.M., Crawford D.C. (2026). Opposing Effects of SARS-CoV-2/COVID-19 Infection and Recombinant Zoster Vaccination on the Risk of Late-Onset Alzheimer Disease. medRxiv.

[25] Rayens E., Sy L.S., Qian L., Wu J., Ackerson B.K., Luo Y., Zheng C., Cheng Y., Vega Daily L.I., Song J. (2025). Adjuvanted Recombinant Zoster Vaccine Is Effective Against Herpes Zoster Ophthalmicus, and Is Associated with Lower Risk of Acute Myocardial Infarction and Stroke in Adults Aged ≥50 Years. Clin. Infect. Dis..

[26] Nguyen R., Desai A. (2026). 26-A-18912-ACC Herpes Zoster Vaccination and Risk of Cardiovascular Events in Patients with Atherosclerotic Cardiovascular Disease. JACC.

[27] Helm M.F., Khoury P.A., Pakchanian H., Raiker R., Maczuga S., Foulke G.T. (2023). Recombinant Zoster Vaccine Reduces 3-Year Cardiovascular Risk: Insights From a Multi-Centered Database. J. Drugs Dermatol..

[28] Parameswaran G.I., Drye A.F., Wattengel B.A., Carter M.T., Doyle K.M., Mergenhagen K.A. (2023). Increased Myocardial Infarction Risk Following Herpes Zoster Infection. Open Forum Infect. Dis..

[29] Kornelius E., Lo S.-C., Huang C.-N., Wang C.-C., Wang Y.-H., Yang Y.-S. (2025). Association of Herpes Zoster Vaccination and Cardiovascular Risk in Patients with Diabetes: Long-Term Insights from a Retrospective Cohort Study. BMJ Open.

[30] Xu X., Ray I., Tang E., Arnold B.F., Acharya N.R. (2025). Protective Effects of Recombinant Zoster Vaccine and Antiviral Therapy Against Cardiovascular Disease Following Herpes Zoster Infection. J. Infect. Dis..

[31] Shih P.-C., Wang S.-I., Leong P.-Y., Huo A.-P., Wei J.C.-C. (2025). Recombinant Zoster Vaccine Reduces All-Cause Mortality, But Not Major Adverse Cardiovascular Events, in Patients with Immune-Mediated Inflammatory Diseases Receiving Janus Kinase Inhibitors: A Large-Scale Real-World Retrospective Cohort Study. J. Med. Virol..

[32] Lin Y.-L., Wang S.-I., Wei J.C.-C. (2025). Effectiveness of Recombinant Zoster Vaccine in Reducing Herpes Zoster Incidence and All-Cause Mortality among Patients with Rheumatoid Arthritis: A Retrospective Cohort Study of 21,046 Individuals from TriNetX U.S. Collaborative Network. EClinicalMedicine.

[33] Kim J.K., Crimmins E.M. (2026). Association between Shingles Vaccination and Slower Biological Aging: Evidence from a US Population-Based Cohort Study. J. Gerontol. Ser. A Biol. Sci. Med. Sci..

[34] Kilic G., Taks E.J.M., Helder L.S., Dulfer E.A., Geckin B., Van Emst L., Lemmers H., Berrè S., Biswas A., Ozturk M. (2026). Age-Related Differences in Immune Responses to Inactivated Influenza and Adjuvanted Recombinant Herpes Zoster Vaccines. JCI Insight.

[35] Welsby I., Detienne S., N’Kuli F., Thomas S., Wouters S., Bechtold V., De Wit D., Gineste R., Reinheckel T., Elouahabi A. (2017). Lysosome-Dependent Activation of Human Dendritic Cells by the Vaccine Adjuvant QS-21. Front. Immunol..

[36] Didierlaurent A.M., Collignon C., Bourguignon P., Wouters S., Fierens K., Fochesato M., Dendouga N., Langlet C., Malissen B., Lambrecht B.N. (2014). Enhancement of Adaptive Immunity by the Human Vaccine Adjuvant AS01 Depends on Activated Dendritic Cells. J. Immunol..

[37] Bosteels C., Fierens K., De Prijck S., Van Moorleghem J., Vanheerswynghels M., De Wolf C., Chalon A., Collignon C., Hammad H., Didierlaurent A.M. (2021). CCR2- and Flt3-Dependent Inflammatory Conventional Type 2 Dendritic Cells Are Necessary for the Induction of Adaptive Immunity by the Human Vaccine Adjuvant System AS01. Front. Immunol..

[38] Cunningham A.L., Heineman T.C., Lal H., Godeaux O., Chlibek R., Hwang S.-J., McElhaney J.E., Vesikari T., Andrews C., Choi W.S. (2018). Immune Responses to a Recombinant Glycoprotein E Herpes Zoster Vaccine in Adults Aged 50 Years or Older. J. Infect. Dis..

[39] Ark R., Allen V., Bechman K., Yang Z., Nagra D., Russell M.D., Galloway J. (2026). Shingles Vaccination in Immune-Mediated Inflammatory Diseases: A Narrative Review. Rheumatology.

[40] Pasoto S.G., Gorayeb T.H.B., Luppino-Assad A.P., Aikawa N.E., Medeiros-Ribeiro A.C., Kupa L.V.K., Borges B.S., Shinjo S.K., De Souza F.H.C., Miossi R. (2026). Immunogenicity, Safety, and Determinants of Recombinant Zoster Vaccine Response in Idiopathic Inflammatory Myopathies: A Phase 4 Randomized Trial. Arthritis Care Res..

[41] Hesse E.M., Shimabukuro T.T., Su J.R., Hibbs B.F., Dooling K.L., Goud R., Lewis P., Ng C.S., Cano M.V. (2019). Postlicensure Safety Surveillance of Recombinant Zoster Vaccine (Shingrix)—United States, October 2017–June 2018. MMWR Morb. Mortal. Wkly. Rep..

[42] Izurieta H.S., Wu X., Forshee R., Lu Y., Sung H.-M., Agger P.E., Chillarige Y., Link-Gelles R., Lufkin B., Wernecke M. (2021). Recombinant Zoster Vaccine (Shingrix): Real-World Effectiveness in the First 2 Years Post-Licensure. Clin. Infect. Dis..

[43] Pang X., Spence O., Parmar N., Wang J., Zhou T., Guo X., Colliou A., Pradeep Sarang S., Yousefi M., Yun H. (2024). A Prospective, Multi-Center Post-Marketing Surveillance Cohort Study to Monitor the Safety of the Recombinant Zoster Vaccine in Chinese Adults ≥50 Years of Age. Hum. Vaccin. Immunother..

[44] Goud R., Lufkin B., Duffy J., Whitaker B., Wong H.-L., Liao J., Lo A.-C., Parulekar S., Agger P., Anderson S.A. (2021). Risk of Guillain-Barré Syndrome Following Recombinant Zoster Vaccine in Medicare Beneficiaries. JAMA Intern. Med..

[45] Kang J.-H., Sheu J.-J., Lin H.-C. (2010). Increased Risk of Guillain-Barré Syndrome Following Recent Herpes Zoster: A Population-Based Study across Taiwan. Clin. Infect. Dis..

[46] Roccatagliata L., Uccelli A., Murialdo A. (2001). Guillain-Barré Syndrome after Reactivation of Varicella-Zoster Virus. N. Engl. J. Med..

[47] Nelson J.C., Ulloa-Pérez E., Yu O., Cook A.J., Jackson M.L., Belongia E.A., Daley M.F., Harpaz R., Kharbanda E.O., Klein N.P. (2023). Active Postlicensure Safety Surveillance for Recombinant Zoster Vaccine Using Electronic Health Record Data. Am. J. Epidemiol..

[48] Xu Y., Li F., Huang L., Mei D., Du P. (2026). Comparative Risk of Guillain-Barré Syndrome Recombinant and Live Zoster Vaccines: A Pharmacovigilance Study Using VAERS and EudraVigilance Database. Immunol. Med..

[49] Cai H., Jia B., Song Z., Wang L., Zhao S. (2025). Real-World Safety of Herpes Zoster Vaccines: A Pharmacovigilance Study Based on the Vaccine Adverse Event Reporting System (May 2006–December 2024). Vaccine.

[50] Shu Y., Cheng W., He X., Huang L., Chen W., Zhang Q. (2025). Post-Marketing Safety Surveillance for the Recombinant Zoster Vaccine (Shingrix), Vaccine Adverse Event Reporting System, United States, October 2017–April 2024. Prev. Med. Rep..

[51] Madani T.A., Khoja A.A., Abuzinadah A.R., Abbas G.M., Alotaibi A.A., Alshehri Z.I., Madani S.T. (2025). Post-Vaccinal Seronegative Autoimmune Encephalitis Following Recombinant Zoster Vaccination in Two Immunocompetent Patients. J. Infect. Chemother..

[52] Wons M.J., Vaghela A., Khalid A., Brooks B.D. (2024). Serious and Progressive Neuropathy Presumably Post-Shingrix Vaccination. Reports.

[53] Asada S., Odera H., Morishita K., Mori S. (2025). Myasthenia Gravis Presenting with Vocal Cord Paralysis as the Initial Symptom Occurring One Week Following Herpes Zoster Vaccination: A Case Report and Review of the Literature. Cureus.

[54] Li H., Xie M., Qiu X., Kang X., Li F., Xu A., Zhang L. (2025). Real-World Study: Analysis of Post-Marketing Safety for Recombinant Zoster Vaccine in Shandong, China. Hum. Vaccin. Immunother..

[55] Wang J., Du J., Liu Y., Xu Y., Han J., Zhang X. (2024). Post-Marketing Surveillance of Adverse Events for the Recombinant Zoster Vaccine Among the Population over 50 Years Old in Hangzhou, China. Vaccines.

[56] Liu G., Zhang X., Wang Y., Wang Y., He P., Yu Q. (2025). Risk of Autoimmune Disease Flares after Recombinant Zoster Vaccine: A Vaccine Safety Analysis Based on the Vaccine Adverse Event Reporting System Database. Hum. Vaccin. Immunother..

[57] Hu G., Varisco D.J., Das S., Middaugh C.R., Gardner F., Ernst R.K., Picking W.L., Picking W.D. (2023). Physicochemical Characterization of Biological and Synthetic Forms of Two Lipid A-Based TLR4 Agonists. Heliyon.

[58] Winthrop K.L., Klaff J., Penn S.K., Liu Y., García C., Mysler E., Wells A.F., Bu X., Fish I., Chen M. (2025). Immunogenicity of Adjuvanted Recombinant Zoster Vaccine in Patients with Rheumatoid Arthritis Treated with Upadacitinib: 60-Week Results from a Randomised Controlled Trial Substudy. RMD Open.

[59] Constenla D., Lonnet G., Aris E., Rk R., Servotte N., Mwakingwe-Omari A., Alsdurf H., Yun H. (2025). Real-World Effectiveness of the Adjuvanted Recombinant Zoster Vaccine in ≥50-Year-Old Adults with Autoimmune Diseases. J. Infect. Dis..

[60] Källmark H., Bergström T., Nagel J., Gullstrand B., Einarsson J.T., Bengtsson A.A., Kapetanovic M.C. (2024). Serologic Immunogenicity and Safety of Herpes Zoster Subunit Vaccine in Patients with Rheumatoid Arthritis Receiving Janus Kinase Inhibitors. Rheumatology.

[61] Hawkins J.A., Curtis J.R., Weinberg A., Siegel S.A.R., Huffstutter J.E., Loveless J., Gharib S., Reddy S., Sinha J., Ridley D. (2026). Safety and Immunogenicity of the Recombinant Zoster Vaccine in Patients with Rheumatoid Arthritis Using Abatacept: A Pilot Multicentre, Double-Blind, Randomised Controlled Trial. Ann. Rheum. Dis..

[62] Baumrin E., Izaguirre N.E., Bausk B., Feeley M.M., Bay C.P., Yang Q., Ho V.T., Baden L.R., Issa N.C. (2021). Safety and Reactogenicity of the Recombinant Zoster Vaccine after Allogeneic Hematopoietic Cell Transplantation. Blood Adv..

[63] Kluberg S.A., Simon A.L., Alam S.M., Peters A., Horgan C., Li D., Moyneur E., Messenger-Jones E., Platt R., McMahill-Walraven C.N. (2024). Risk of Incident Gout Following Exposure to Recombinant Zoster Vaccine in US Adults Aged ≥50 Years. Semin. Arthritis Rheum..

[64] Morrone G.M., Sandri S., Vernero M., Armandi A., Caviglia G.P., Ribaldone D.G. (2026). Real-World Safety and Herpes Zoster Outcomes After Recombinant Zoster Vaccination in Inflammatory Bowel Disease. J. Clin. Med..

[65] Valentini S., Sota J., Fineschi I., Conticini E., Garcia-Gonzalez E., D’Ignazio E., Bardelli M., Gentileschi S., Fabbroni M., Bellisai F. (2025). Effectiveness and Safety of Recombinant Zoster Vaccine in Rheumatic Diseases: Real-World Evidence from a Single-Centre Italian Cohort. Vaccines.

[66] Aikawa N.E., Medeiros-Ribeiro A.C., Pasoto S.G., Kupa L.V.K., Seguro L.P.C., Luppino-Assad A.P., Borba E.F., Saad C.G.S., Yuki E.F.N., Andrade D.C.O. (2026). Recombinant Herpes Zoster Vaccine in Patients with Autoimmune Rheumatic Diseases in Brazil: A Double-Blind, Randomised, Placebo-Controlled, Phase 4, Non-Inferiority Study. Lancet Rheumatol..

[67] Luppino-Assad A.P., Mello R.P., Medeiros-Ribeiro A.C., Aikawa N.E., Pasoto S.G., Praxedes A.C., Kupa L.V., Miossi R., Sampaio-Barros P.D., Borba E.F. (2026). Prospective Evaluation of Recombinant Herpes Zoster Vaccine in SSc: Immunogenicity, Safety and Disease Outcomes. Rheumatology.

[68] Dagnew A.F., Rausch D., Hervé C., Zahaf T., Levin M.J., Schuind A., ZOE-50/70 Study Group (2021). Efficacy and Serious Adverse Events Profile of the Adjuvanted Recombinant Zoster Vaccine in Adults with Pre-Existing Potential Immune-Mediated Diseases: A Pooled Post Hoc Analysis on Two Parallel Randomized Trials. Rheumatology.

[69] Lenfant T., Jin Y., Kirchner E., Hajj-Ali R.A., Calabrese L.H., Calabrese C. (2021). Safety of Recombinant Zoster Vaccine: A Retrospective Study of 622 Rheumatology Patients. Rheumatology.

[70] Yoon J.G., Nham E., Choi Y.J., Choi W.S., Choi M.J., Seo Y.B., Lee J., Hyun H., Heo J.Y., Noh J.Y. (2026). Comparison of Vaccine Adverse Events Between Zoster Vaccine Live and Recombinant Zoster Vaccine: A Prospective Multicenter Survey. J. Korean Med. Sci..

[71] Muttucumaru R., Lau C.L., Leeb A., Mills D.J., Wood N., Furuya-Kanamori L. (2024). Post-Marketing Surveillance of 10,392 Herpes Zoster Vaccines Doses in Australia. Vaccine.

[72] Levine K.S., Leonard H.L., Blauwendraat C., Iwaki H., Johnson N., Bandres-Ciga S., Ferrucci L., Faghri F., Singleton A.B., Nalls M.A. (2023). Virus Exposure and Neurodegenerative Disease Risk across National Biobanks. Neuron.

[73] Itzhaki R.F., Lin W.R., Shang D., Wilcock G.K., Faragher B., Jamieson G.A. (1997). Herpes Simplex Virus Type 1 in Brain and Risk of Alzheimer’s Disease. Lancet.

[74] Silver B., Nagel M.A., Mahalingam R., Cohrs R., Schmid D.S., Gilden D. (2012). Varicella Zoster Virus Vasculopathy: A Treatable Form of Rapidly Progressive Multi-Infarct Dementia after 2 Years’ Duration. J. Neurol. Sci..

[75] Gilden D., Grose C., White T., Nagae L., Hendricks R.L., Cohrs R.J., Nagel M.A. (2016). Successful Antiviral Treatment after 6years of Chronic Progressive Neurological Disease Attributed to VZV Brain Infection. J. Neurol. Sci..

[76] Bubak A.N., Como C.N., Hassell J.E., Mescher T., Frietze S.E., Niemeyer C.S., Cohrs R.J., Nagel M.A. (2022). Targeted RNA Sequencing of VZV-Infected Brain Vascular Adventitial Fibroblasts Indicates That Amyloid May Be Involved in VZV Vasculopathy. Neurol. Neuroimmunol. Neuroinflamm..

[77] Nagel M.A., Cohrs R.J., Mahalingam R., Wellish M.C., Forghani B., Schiller A., Safdieh J.E., Kamenkovich E., Ostrow L.W., Levy M. (2008). The Varicella Zoster Virus Vasculopathies: Clinical, CSF, Imaging, and Virologic Features. Neurology.

[78] Nagel M.A., Traktinskiy I., Azarkh Y., Kleinschmidt-DeMasters B., Hedley-Whyte T., Russman A., VanEgmond E.M., Stenmark K., Frid M., Mahalingam R. (2011). Varicella Zoster Virus Vasculopathy: Analysis of Virus-Infected Arteries. Neurology.

[79] Nagel M.A., Traktinskiy I., Stenmark K.R., Frid M.G., Choe A., Gilden D. (2013). Varicella-Zoster Virus Vasculopathy: Immune Characteristics of Virus-Infected Arteries. Neurology.

[80] Nagel M.A., Bubak A.N. (2018). Varicella Zoster Virus Vasculopathy. J. Infect. Dis..

[81] Attems J., Jellinger K.A. (2014). The Overlap between Vascular Disease and Alzheimer’s Disease--Lessons from Pathology. BMC Med..

[82] Boyle P.A., Yu L., Nag S., Leurgans S., Wilson R.S., Bennett D.A., Schneider J.A. (2015). Cerebral Amyloid Angiopathy and Cognitive Outcomes in Community-Based Older Persons. Neurology.

[83] Devanand D.P. (2018). Viral Hypothesis and Antiviral Treatment in Alzheimer’s Disease. Curr. Neurol. Neurosci. Rep..

[84] Itzhaki R.F. (2021). Overwhelming Evidence for a Major Role for Herpes Simplex Virus Type 1 (HSV1) in Alzheimer’s Disease (AD); Underwhelming Evidence Against. Vaccines.

[85] Cairns D.M., Itzhaki R.F., Kaplan D.L. (2022). Potential Involvement of Varicella Zoster Virus in Alzheimer’s Disease via Reactivation of Quiescent Herpes Simplex Virus Type 1. J. Alzheimers Dis..

[86] Schmidt S.A.J., Veres K., Sørensen H.T., Obel N., Henderson V.W. (2022). Incident Herpes Zoster and Risk of Dementia: A Population-Based Danish Cohort Study. Neurology.

[87] McGirr A., Widenmaier R., Curran D., Espié E., Mrkvan T., Oostvogels L., Simone B., McElhaney J.E., Burnett H., Haeussler K. (2019). The Comparative Efficacy and Safety of Herpes Zoster Vaccines: A Network Meta-Analysis. Vaccine.

[88] Gilden D., Cohrs R.J., Mahalingam R., Nagel M.A. (2009). Varicella Zoster Virus Vasculopathies: Diverse Clinical Manifestations, Laboratory Features, Pathogenesis, and Treatment. Lancet Neurol..

[89] Parameswaran G.I., Wattengel B.A., Chua H.C., Swiderek J., Fuchs T., Carter M.T., Goode L., Doyle K., Mergenhagen K.A. (2023). Increased Stroke Risk Following Herpes Zoster Infection and Protection with Zoster Vaccine. Clin. Infect. Dis..

[90] (2024). GBD 2021 Stroke Risk Factor Collaborators Global, Regional, and National Burden of Stroke and Its Risk Factors, 1990-2021: A Systematic Analysis for the Global Burden of Disease Study 2021. Lancet Neurol..

[91] Furman D., Campisi J., Verdin E., Carrera-Bastos P., Targ S., Franceschi C., Ferrucci L., Gilroy D.W., Fasano A., Miller G.W. (2019). Chronic Inflammation in the Etiology of Disease across the Life Span. Nat. Med..

[92] Stepanova M., Rodriguez E., Birerdinc A., Baranova A. (2015). Age-Independent Rise of Inflammatory Scores May Contribute to Accelerated Aging in Multi-Morbidity. Oncotarget.

[93] Xu W., Liang Y., Lin Z. (2021). Association Between Neutrophil-Lymphocyte Ratio and Frailty: The Chinese Longitudinal Healthy Longevity Survey. Front. Med..

[94] Detienne S., Welsby I., Collignon C., Wouters S., Coccia M., Delhaye S., Van Maele L., Thomas S., Swertvaegher M., Detavernier A. (2016). Central Role of CD169^+^ Lymph Node Resident Macrophages in the Adjuvanticity of the QS-21 Component of AS01. Sci. Rep..

[95] Coccia M., Collignon C., Hervé C., Chalon A., Welsby I., Detienne S., van Helden M.J., Dutta S., Genito C.J., Waters N.C. (2017). Cellular and Molecular Synergy in AS01-Adjuvanted Vaccines Results in an Early IFNγ Response Promoting Vaccine Immunogenicity. npj Vaccines.

[96] Ren W., Zhao L., Sun Y., Wang X., Shi X. (2023). HMGB1 and Toll-like Receptors: Potential Therapeutic Targets in Autoimmune Diseases. Mol. Med..

[97] Li J., Wang X., Zhang F., Yin H. (2013). Toll-like Receptors as Therapeutic Targets for Autoimmune Connective Tissue Diseases. Pharmacol. Ther..

[98] Liu B., Li A., Liu Y., Zhou X., Xu J., Zuo X., Xue K., Cui Y. (2024). Transcobalamin 2 Orchestrates Monocyte Proliferation and TLR4-Driven Inflammation in Systemic Lupus Erythematosus via Folate One-Carbon Metabolism. Front. Immunol..

[99] Feng Z., Meng F., Huo F., Zhu Y., Qin Y., Gui Y., Zhang H., Lin P., He Q., Li Y. (2024). Inhibition of Ferroptosis Rescues M2 Macrophages and Alleviates Arthritis by Suppressing the HMGB1/TLR4/STAT3 Axis in M1 Macrophages. Redox Biol..

[100] Bechtold V., Smolen K.K., Burny W., de Angelis S.P., Delandre S., Essaghir A., Marchant A., Ndour C., Taton M., van der Most R. (2024). Functional and Epigenetic Changes in Monocytes from Adults Immunized with an AS01-Adjuvanted Vaccine. Sci. Transl. Med..

[101] National Library of Medicine (2026). AREXVY.

[102] Taquet M., Todd J.A., Harrison P.J. (2025). Lower Risk of Dementia with AS01-Adjuvanted Vaccination against Shingles and Respiratory Syncytial Virus Infections. npj Vaccines.

[103] Singer D., Steffens A., La E.M., Buikema A.R., Horn E.K., Theiss-Nyland K., Fonseca M.J., Gerber S.I., Kawai A., Johnson M.G. (2026). Effectiveness of the Adjuvanted Respiratory Syncytial Virus Prefusion F3 Vaccine in Preventing Major Adverse Cardiovascular Events and Severe Exacerbations of COPD and Asthma Among US Adults ≥ 60 Years of Age. CHEST.

[104] European Medicines Agency (2015). Mosquirix: EPAR Summary for the Public.

[105] Heidenreich P., Bhatt A., Nazir N.T., Schaffner W., Vardeny O. (2025). 2025 Concise Clinical Guidance: An ACC Expert Consensus Statement on Adult Immunizations as Part of Cardiovascular Care. JACC.

[106] Heidecker B., Libby P., Vassiliou V.S., Roubille F., Vardeny O., Hassager C., Gatzoulis M.A., Mamas M.A., Cooper L.T., Schoenrath F. (2025). Vaccination as a New Form of Cardiovascular Prevention: A European Society of Cardiology Clinical Consensus Statement. Eur. Heart J..

